# Hypoglycemic Risk Factors Among Hospitalized Patients with Type 2 Diabetes Mellitus at King Abdulaziz Medical City, Jeddah

**DOI:** 10.7759/cureus.6742

**Published:** 2020-01-22

**Authors:** Erada M Alghamdi, Laila A Alghubayshi, Reem A Alshamrani, Rawan A Alnajashi, Ebtihal A Alamoudi, Amani M Aljabarti, Hawazen A Zarif

**Affiliations:** 1 Internal Medicine, College of Medicine, King Saud Bin Abdulaziz University for Health Sciences, King Abdullah Medical Research Center, Ministry of the National Guard, Jeddah, SAU; 2 Internal Medicine, King Abdulaziz University, Jeddah, SAU; 3 Medicine / Endocrinology, Ministry of the National Guard - Health Affairs, King Abdullah International Medical Research Center, Jeddah, SAU

**Keywords:** hypoglycemia, hospitalized diabetic patients, hypoglycemia risk factors

## Abstract

Background

Hypoglycemia is a pathological condition in which the serum glucose level measures less than 3.0 mmol/L. It is a well-known complication in patients with diabetes mellitus. Age, body weight, gender, insulin usage, nutritional therapy, body mass index (BMI), the presence of diabetes complications, intensive care unit admission, and infection were reported as possible risk factors that may increase the risk of hypoglycemia. Therefore, this study aimed to analyze predisposing factors for hypoglycemia among hospitalized patients with type 2 diabetes in King Abdulaziz Medical City.

Method

This is a retrospective, case-control study design. The study included 326 hospitalized patients with type 2 diabetes; 152 experienced hypoglycemia (blood glucose <3.9) at least once during hospitalization and have been compared to 174 in the non-hypoglycemic group (blood glucose ≥3.9). Data were extracted from their electronic medical records (EMRs).

Results

This study reported that patients with lower BMI (28.80 ± 7 versus 31.20 ± 12.93) experienced hypoglycemia (P-value 0.044). Those hospitalized with infections or had acquired infections or required intensive care unit (ICU) admission during hospitalization had a higher risk to develop hypoglycemia (P-value 0.005, 0.003, and <0.001, respectively). Moreover, the use of multiple doses of insulin therapy or basal-plus insulin therapy was associated with a higher risk of hypoglycemia (P-value 0.012 and 0.028, respectively). Those on supplemental insulin were less likely to develop hypoglycemia (P-value <0.001). Patients on oral feeding had a lower chance of having a hypoglycemic attack (P-value 0.002) while those on tube feeding had double the odds (OR=2.37).

Conclusions

Infection, intensive care unit admission, lower body mass index, insulin regimen and nutritional therapy (enteral feeding and nothing-per-mouth (NPO)) were correlated with an elevated risk of having hypoglycemia in hospitalized patients with type 2 diabetes mellitus.

## Introduction

Diabetes mellitus (DM) is a metabolic disease, in which the serum glucose level is elevated due to a problem in insulin synthesis and/or its function [1]. The mechanism for which DM develops a range from being an autoimmune process leading to the loss of the beta cells of the pancreas, resulting in deficient insulin secretion, to cellular irregularities that lead to insulin resistance [1]. DM is a multinational health problem that needs lifelong intervention. According to the World Health Organization (WHO), in the past few years, the incidence of DM had increased by up to 8.5% in the past few years, type 2 accounts for the majority of cases [2]. Saudi Arabia ranks second and seventh in the prevalence of DM in the Middle East and worldwide, respectively [3]. Furthermore, the prevalence of DM was estimated to be 34.1% in males and 27.6% in females in the Saudi population [4]. Untreated and uncontrolled DM has always been associated with several complications. This includes macrovascular complications, such as peripheral vascular disease, cerebrovascular and cardiovascular, and macrovascular such as neuropathy, retinopathy, and nephropathy [5-6]. The rate of macrovascular and microvascular DM-associated complications in the Middle East was reported to be 28.7% and 65.8%, respectively, and more than half (53.4%) of the micro-complications was found to be neuropathy related [7].

Diabetes was found to increase the risk of hospital admission [8]. A recent study conducted in Saudi Arabia revealed that DM was one of the most common comorbid diseases in hospitalized patients with a percentage of 39.2% [9]. The American Diabetes Association (ADA) recommended starting treatment with insulin when the serum glucose level is persistently high, more than or equals to 180 mg/dL (10.0 mmol/L) [10]. With insulin therapy, the target blood glucose for the majority of in-patients should range from 140-180 mg/dL (7.8-10 mmol/L). In some groups of patients, a more strict glycemic goal, such as blood glucose ranges from 110-140 mg/dL, might be applicable [10]. Furthermore, hypoglycemia, which is one of the common and significant complications of DM, has been associated with adverse clinical outcomes [11-12]. In the United States, the prevalence of hypoglycemia in hospitalized patients with intensive care unit (ICU) and without ICU admission were 6.3% and 5.3%, respectively [13]. Various factors have been recognized as a risk for hypoglycemia such as age, cardiovascular and kidney diseases, and infection [14-15]. Also, female gender, low body weight, and insulin use have been linked in previous studies as the risk factors of severe hypoglycemia [16-17]. A hypoglycemic event among the population with DM may contribute to the high risk of mortality and prolonged hospitalization [18]. Therefore, the primary goal of the study was to determine hypoglycaemic risk factors among hospitalized patients with type 2 DM (T2DM), who were admitted for medical or surgical health problems at King Abdulaziz Medical City.

## Materials and methods

Study population

Patients with type 2 diabetes, aged 18 years and above, who were hospitalized for three days or more between the period of July 1, 2016, to June 30, 2017, were eligible to be included. Hypoglycemia subjects must have had at least one hypoglycemic attack during hospitalization, whereas the control group must have had no hypoglycemic attack. Moreover, pregnant women with or without gestational diabetes, type 1 DM (T1DM), and individuals who admitted to the ICU upon presentation were excluded from the study. Patients diagnosed with hypoglycemia, hyperosmolar hyperglycemic state, or diabetic ketoacidosis were also excluded. The study was approved by the Research Committee, College of Medicine at King Abdulaziz University for Health Sciences, as well as by the Institutional Review Board (IRB) of King Abdullah International Medical Research Centre.

Study design

This is a case-control study design, where the study sample was obtained retrospectively from the lab records of blood glucose results. The values of capillary glucose were used to identify the groups. Individuals with blood sugar less than 3.9 mmol/L (70 mg/dL) were included in the case group, whereas those who had readings equal to or greater than 3.9 mmol/L (70 mg/dL) were included in the control group. The subjects of each group were randomly selected using the Microsoft Excel program (Microsoft Corporation, Redmond, Washington). An electronic medical record (BestCare; Seoul, Korea) was used to extract the required information.

Measurements

The electronic charts of the study subjects were reviewed, and the necessary information was obtained retrospectively and abstracted to a standardized collection Excel sheet. Data collected included gender, age, body mass index (BMI), diabetes complications, presence of cancer, number of hospital admissions during the study period, length of hospital stay, and laboratory tests. It also contained a diagnosis, hospital complications, antidiabetic treatment, and type of nutritional therapy.

The complications of diabetes included both microvascular (retinopathy, nephropathy, and neuropathy) and macrovascular (peripheral artery disease, ischemic heart disease, and stroke). Laboratory studies abstracted were hemoglobin A1c (%), serum creatinine (μmol/L), and blood culture. Hospital complications involved the presence of in-hospital infections (cellulitis, urinary tract infection, pneumonia, and sepsis) and the need for ICU admission during hospitalization. Diabetic medications involved insulin (supplemental: sliding scale or corrective dose; basal: glargine, detemir, neutral protamine Hagedorn (NPH); basal plus: basal plus supplemental; conventional: any type of insulin less than three doses per day; MDI: multiple doses insulin more than three doses per day; and other regimen) and oral hypoglycemic agents (metformin, sulfonylurea, combination of any two types, and others). Nutritional therapy was any of the three: oral, tube feeding, or nothing per mouth (NPO). Acute infections defined as the occurrence of an infection at the time of hospitalization, and it included sepsis, pneumonia, urinary tract infection, and others. 

Data analysis

The average number of hospitalized diabetic patients at King Abdulaziz Medical City was found to be 5436 yearly. To detect the difference of the risk factors between the case and control groups (P1=0.15 and P2=0.5, delta=0.35, 0.05 alpha, and 80% power), we needed to recruit 300 patients, 150 patients in each group. Data were analyzed using SPSS statistical software (version 25.0; IBM Corp, Armonk, NY), and a p-value of less than 0.05 was considered statistically significant. Frequencies and percentages were used to express categorical data and tested with the Chi-square test. Mean ± standard deviation or median and interquartile range (IQR) were used to expressed quantitative data, as appropriate. The student t-test or Mann-Whitney test was used to determine the differences between the groups. Multivariate logistic regression analysis was used to control possible cofounder variables and to identify the most predictive factors associated with hypoglycemia. The results were expressed as odds ratio (OR) and 95% CIs.

## Results

The baseline characteristics of the study are presented in Table 1. The study included 326 patients, with 152 (46.6%) subjects who had hypoglycemia. The male proportion was 59.2% in the hypoglycemic group and 56.9% in the control group. In the hypoglycemic group, the mean age was 66.98 ± 12.06 years and the mean BMI was 28.8 ± 7.00 kg/m^2^.

**Table 1:** Baseline characteristics of hospitalized patients with type 2 diabetes a. BMI: Body Mass Index, b. HgbA1c: Glycated Hemoglobin A1c

	Case Group	Control Group	OR	P-value	95% CI
Sample, N (%)	152 (46.6)	174 (53.4)			
Age (years), Mean ± SD	66.98 ± 12.06	65.96 ± 13.24		0.470	
Male	90 (59.2)	99 (56.9)		0.673	0.585 – 1.414
Female	62 (40.8)	75 (43.1)			
BMI^a ^kg/m^2^, Mean ± SD	28.80 ± 7.00	31.20 ± 12.93		0.044	0.064 – 4.722
Presence of DM complications	116 (76.3)	94 (54)	2.742	<0.001	1.700 – 4.424
- Neuropathy, N (%)	16 (10.5)	28 (16.1)	0.613	0.142	0.318 – 1.184
- Nephropathy, N (%)	53 (34.9)	44 (25.3)	1.582	0.059	0.981 – 2.550
- Retinopathy, N (%)	18 (11.8)	23 (13.2)	0.882	0.709	0.456 – 1.705
- Stroke, N (%)	51 (33.6)	35 (20.1)	2.005	0.006	1.215 – 3.309
- Ischemic heart disease, N (%)	53 (34.9)	58 (33.3)	1.071	0.770	0.677 – 1.694
- Diabetic foot, N (%)	23 (15.1)	8 (4.6)	3.700	0.001	1.602 – 8.542
- Amputation, N (%)	13 (8.6)	7 (4)	2.231	0.089	0.866 – 5.746
Malignancy	31 (20.5)	59 (33.9)	0.504	0.007	0.304 – 0.834
No of hospital admission, median (IQR)	2 (2)	1 (1)		0.003	
Length of stay, median (IQR)	15 (21)	8 (10)		<0.001	
HgbA1c^b ^% Mean ± SD	7.96 ± 1.79	8.06 ± 2.22		0.724	-0.440 – 0.632
Creatinine umol/L, median (IQR)	86.0 (90.0)	81.0 (56.5)		0.844	124.95 – 162.76

The presence of DM complications was 76.3% in the case group and 54% in the control, with an odds ratio of 2.74. Among all micro and macrovascular complications, only stroke and diabetic foot were significant (OR=2.00 and 3.70, respectively).

The median number of hospital admissions was two, 15 days was the median length of hospitalization while 28 days was the median number for intensive care unit (ICU) admission, mean hemoglobin A1c was 7.96 with SD 1.79, and median serum creatinine was 86 µmol/L (IQR 60).

The diagnosis of admission and hospital complications were demonstrated in Table 2. Infection as a cause of admission was statistically significant (P-value=0.005, OR=2.12). Moreover, the occurrence of infection during the hospital stay in patients with low blood sugar was 82% (P-value=0.003, OR=1.96). Among all types of infections, pneumonia and sepsis were statistically significant (P-value=0.012, OR=2.17; P-value=0.013, OR=2.35, respectively).

**Table 2:** Primary diagnosis and hospital complications of the two study groups a. UTI: Urinary Tract Infection, b. ICU: Intensive Care Unit

	Case Group	Control Group	OR	P-value	95% CI
Diagnosis on admission					
- Infection, N (%)	44 (28.9)	28 (16.1)	2.124	0.005	1.244 – 3.628
- Cardiovascular, N (%)	19 (12.5)	20 (11.5)	1.100	0.780	0.563 – 2.148
- Neurological, N (%)	6 (3.9)	14 (8)	0.470	0.124	0.176 – 1.254
- Malignancy, N (%)	27 (17.8)	43 (24.7)	0.658	0.127	0.383 – 1.129
- Pulmonary, N (%)	8 (5.3)	7 (4)	1.325	0.594	0.469 – 3.745
- Other, N (%)	48 (31.6)	62 (35.6)	0.834	0.440	0.525 – 1.323
Hospital complications					
- In-hospital Infection, N (%)	82 (53.9)	65 (37.4)	1.964	0.003	1.262 – 3.059
- Cellulitis	9 (5.9)	6 (3.4)	1.762	0.288	0.613 – 5.070
- UTI^c^	23 (15.1)	30 (17.2)	0.856	0.607	0.473 – 1.548
- Pneumonia	32 (21.1)	19 (10.9)	2.175	0.012	1.175 – 4.026
- Sepsis	26 (17.1)	14 (8)	2.358	0.013	1.182 – 4.704
- Other infections	39 (25.7)	15 (8.6)	3.658	<0.001	1.924 – 6.955
- ICU^b^ admission, N (%)	28 (18.4)	8 (4.6)	4.685	<0.001	2.065 – 10.633

Table 3 shows the distribution of diabetic medications and nutritional therapy among the study group. A total of 281 patients (90.1% hypoglycemia cases versus 82.8% in control) were given insulin during their hospital stay (P-value 0.054). The results of the sub-grouping analysis of insulin types were supplemental (P-value<0.001, OR=0.36.), basal (P-value=0.028, OR=1.94), conventional (P-value=0.169, OR=1.74), and MDI (P-value=0.012, OR=1.81). A total of 45 patients (21 cases, 24 control) of the study sample were on oral hypoglycemic agents (OHA) during their admission. Regarding hypoglycemic subjects, the analysis of nutritional therapy showed that oral feeding was 71.7% (OR=0.42), tube feeding was 13.8% (OR=2.37), and NPO was 14.5% (OR=1.93).

**Table 3:** Distribution of diabetic medications and nutritional therapy in hospitalized patients who developed hypoglycemia versus patients without hypoglycemia a. Supplemental: Sliding Scale or Corrective Dose Insulin, b. Basal: Glargine, NPH, or Detemir Insulin, c. Plus sign (+): supplemental, d. Conventional: Less than 3 Doses per Day, e. MDI: Multiple Dose Insulin, f. OHA: Oral Hypoglycemic Agent, g. NGT: Nasogastric Tube, h. PEG: Percutaneous Endoscopic Gastrostomy Tube, i. NPO: Nothing-per-Oral

	Case Group	Control Group	OR	P-value	95% CI
Insulin use, N (%)	137 (90.1)	144 (82.8)	1.903	0.054	0.981 – 3.691
- Supplemental^a^	22 (14.5)	55 (31.6)	0.366	<0.001	0.211 – 0.637
- Basal^b^/Basal+^c^	32 (21.1)	21 (12.1)	1.943	0.028	1.066 – 3.540
- Conventional^d^/Conventional+	16 (10.5)	11 (6.3)	1.743	0.169	0.783 – 3.883
- MDI^e^/MDI+	60 (39.5)	46 (26.4)	1.815	0.012	1.136 – 2.899
- Other	7 (4.6)	11 (6.3)	0.715	0.498	0.270 – 1.894
OHA^f^ use, N (%)	21 (13.8)	24 (13.8)	1.002	0.995	0.533 – 1.883
Nutritional therapy					
- Oral, N (%)	109 (71.7)	149 (85.61)	0.425	0.002	0.245 – 0.738
- NGT^g^/PEG^h^ tube, N (%)	21 (13.8)	11 (6.3)	2.375	0.023	1.106 – 5.104
- NPO^i^, N (%)	22 (14.5)	14 (8)	1.934	0.065	0.952 – 3.930

Multivariate regression analysis was used to identify the most predictive risk factors to develop hypoglycemia (Table 4). The results showed that BMI, infection (as an admission diagnosis), ICU admission, nutritional therapy, and insulin use were significant predictors (P-values 0.025, 0.012, <0.001, 0.021, and 0.030, respectively).

**Table 4:** Multiple logistic regression analysis for the most predictive factors of hypoglycemia a. BMI: Body Mass Index, b. ICU: Intensive Care Unit

Factors	P-value
Gender	0.568
Age (years)	0.535
Insulin Use	0.030
BMI^a^ (kg/m^2^)	0.025
Nutritional Therapy	0.021
Admission with Infection	0.012
ICU^b^ Admission	<0.001

## Discussion

The current study aimed to assess the risk factors of developing hypoglycemia in hospitalized patients with type 2 diabetes (T2DM). The study included 326 patients; 46.6% with a hypoglycemic attack during their hospital stay (defined as blood glucose less than 3.9 mmol/L). The clinical characteristics of the study subjects described in this study were similar among the groups.

Increased age is a known risk factor for developing hypoglycemia, which could be related to an increase in comorbidities. However, no link was observed between the group in the present study (P-value=0.470), which is similar to a study done by Kagansky et al. [19]. We also found that there was no association between gender and hypoglycemia, although other studies reported that females had a high chance of developing hypoglycemia as compared to males [19-20]. 

A study conducted by Elliott et al. reported that low body weight was a strong risk factor for hypoglycemia, which is similar to our findings [21]. However, in our study, the greater number of the patients were overweight in the hypoglycemic group (BMI = 28.80 ± 7.00) and obese in the control group (BMI=31.20 ± 12.93).

Similar to previous studies reported by Kagansky et al. and Lin et al., we found that patients who were admitted with an infection had double the risks of developing hypoglycemia during admission (OR, 2.124; 95%CI, 1.244 to 3.628) [19,22]. Otherwise, the analysis of patients who were admitted due to other medical or surgical causes was comparable. Furthermore, patients who were admitted to the ICU during their hospital course had a four times higher risk of hypoglycemia (OR, 4.685; 95% CI, 2.065 to 10.633). Arabi et al. stated that “longer ICU length of stay (LOS) is associated with more chances of developing hypoglycemia” [20]. We found that patients who developed hypoglycemia during admission had a more extended hospital stay (P <0.001) than those who did not; this shows similarity to the result of Brodovicz et al. [18]. This could be explained by the severity of illness in those who had developed hypoglycemia.

Nutritional therapy might have a crucial role in developing hypoglycemia. In the present study, we found patients on oral feeding had lower odds of developing hypoglycemic events in the hospital (OR, 0.42; 95% CI, 0.24 to 0.73), and those who were NPO had approximately double the odds and even more in those on tube feeds (OR 2.375; 95% CI 1.106 - 5.104). Hence, oral feeding could be a preventive factor of low blood sugar. Therefore, in hospitalized patients, the anti-diabetic medication should be adjusted according to the nutritional status of patients.

Previous studies mentioned that there is an association between hypoglycemia and the presence of comorbid conditions [23-24]. An association was found between the diagnosis of cancer and the risk of hypoglycemia in the present study (P-value 0.007). Patients with malignancy have lower odds of developing hypoglycemia (OR, 0.504). This might be explained by loose glycaemic control in those patients.

The current study shows that diabetic complications were a strong predictor for hypoglycemia (OR, 2.74; 95% CI, 1.70 to 4.42). It is well-established from different published studies that the micro and macro complications of diabetes, in addition to infection, are considered predisposing variables for severe hypoglycemia [20,25]. In this study, patients with diabetic foot were three times more likely to have hypoglycemia (OR, 3.70; 95% CI, 1.60 to 8.54). The risk for hypoglycemia was shown to be increased in patients with a history of cardiovascular diseases (CVD) and renal disease, but no significance was observed among study groups in this study [21,23]. Retinopathy, neuropathy, and amputation were also reported to be associated with severe hypoglycemia [22,23]. However, our findings showed no increased risk of hypoglycemia among patients with these complications. Although previous studies found no association between stroke and hypoglycemia, our analysis showed that hypoglycemic events increased two folds in T2DM patients with a previous history of stroke (OR, 2.00; 95% CI, 1.21 to 3.30) [26].

Insulin therapy has always been linked to causing hypoglycemia in the diabetic population. Rubin et al. reported a significantly increased risk of hypoglycemia with those who are not receiving a sliding scale as their insulin regimen [27]. This matches this studies' results that patients on supplemental insulin (sliding scale or corrective dose) during the hospitalization had a lower chance to have hypoglycemia (OR, 0.36; 95% CI, 0.21 to 0.63), whereas those who were given basal insulin (OR, 1.94; 95% CI, 1.06 to 3.54) or were treated by MDI (OR 1.815; CI 1.136 - 2.899) had almost double the risk.

The use of oral diabetic agents has been found to elevate the risk of hypoglycemia as established by many studies [19,27-28]. This was not observed in the current study, as we observed no differences between the study groups regarding the use of oral hypoglycemic medications on developing hypoglycemia. However, only a small number of study subjects (21 cases and 24 control) were taking oral hypoglycemic agents (OHA) during hospitalization, as the majority of patients were shifted to insulin therapy on admission.

Our study has some limitations. As it was a retrospective chart review, some data were not available in the EMRs of the patients. In addition, as our focus was on hypoglycemia, although some insulin regimens have a less hypoglycemic effect, this could be at the expense of having hyperglycemia, which is beyond the scope of this research.

## Conclusions

In conclusion, we found that the insulin regimen used, admission with an infection, ICU admission, lower BMI, and nutritional therapy were independent predictive factors of hypoglycemia among hospitalized patients with T2DM. Patients who are hospitalized with those risk factors need regular monitoring of serum glucose more often than other patients to reduce the risk of developing hypoglycemia. Further studies need to be conducted to identify the optimum insulin regimen to use and the frequency of blood glucose monitoring to avoid hypoglycemia in patients with those risk factors.
